# Overcoming the UCB HSCs –Derived NK cells Dysfunction through Harnessing RAS/MAPK, IGF-1R and TGF-β Signaling Pathways

**DOI:** 10.1186/s12935-021-01983-z

**Published:** 2021-06-07

**Authors:** Alireza Shokouhifar, Gholamreza Anani Sarab, Mahboubeh Yazdanifar, Mohammad Fereidouni, Masoumeh Nouri, Marzieh Ebrahimi

**Affiliations:** 1 Department of Molecular Medicine, Genomic Research Center, Birjand University of Medical Sciences, Birjand, Iran; 2 Cellular & Molecular Research Center, Birjand University of Medical Sciences, Birjand, Iran; 3 Department of Stem Cells and Developmental Biology, Cell Science Research Center, Royan Institute for Stem Cell Biology and Technology, ACECR, Tehran, Iran; 4 Stem Cell Transplantation and Regenerative Medicine, Department of Pediatrics, Stanford University School of Medicine, Palo Alto, CA USA; 5 R&D Department, Royan Stem Cell Technology Co, Tehran, Iran

**Keywords:** Umbilical cord blood, Natural killer cells, Differentiation, Cytotoxicity, Cancer immunotherapy

## Abstract

**Background:**

The natural killer (NK) cells differentiated from umbilical cord blood (UCB) hematopoietic stem cells (HSCs) may be more suitable for cell-based immunotherapy compared to the NK cells from adult donors. This is due to the possibility to choose alloreactive donors and potentially more robust in vivo expansion. However, the cytotoxicity of UCB-HSC-derived NK cells against cancer cells might be suboptimal. To overcome this obstacle, we attempted to generate NK cells with potent antitumor activity by targeting RAS/MAPK, IGF-1R and TGF-β signaling pathways using IL-15, IGF-1 and SIS3 respectively.

**Methods:**

The CD34 + cells were isolated from human UCB mononuclear cells through magnetic activation cell sorting (MACS) with purity of (≥ 90%) and were subjected to differentiate into NK cells. After 21 days of induction with SFTG36 (SCF, FLt-3L, TPO, GM-CSF, IL-3 and IL-6), IS721 (IGF-1, SIS3, IL-7 and IL-21) and IL-15/Hsp70 media, NK cells phenotypes were studied and their cytotoxicity against K562 human erythroleukemia cells and SKOV3 ovarian carcinoma cells was analyzed.

**Results:**

The NK cells induced in SFTG36/IS721 medium were selected for activation due to their higher expression of CD56 + 16 + CD3 −  (93.23% ± 0.75) and mean fluorescence intensity (MFI) of NKG2D + (168.66 ± 20.00) and also a higher fold expansion potential (11.893 ± 1.712) compared to the other groups. These cells once activated with IL-15, demonstrated a higher cytotoxicity against K562 (≥ 90%; P ≤ 0.001) and SKOV3 tumor cells (≥ 65%; P ≤ 0.001) compared to IL-15/Hsp70-activated NK cells.

**Conclusions:**

The differentiation of ex vivo expanded CD34 + cells through manipulation of RAS/MAPK, IGF-1R and TGF-β signaling pathways is an efficient approach for generating functional NK cells that can be used for cancer immunotherapy.

**Supplementary Information:**

The online version contains supplementary material available at 10.1186/s12935-021-01983-z.

## Background

Immune cell-based therapy aims at harnessing the patient’s own immune system to fight cancer and to obtain long-term responses in patients [1, 2]. Among various components of the immune system, natural killer (NK) cells of the innate immune system lymphocytes are able to kill tumor and virus-infected cells without prior sensitization to antigens [3, 4]. For this, many efforts have been made to apply the NK cells derived from peripheral blood to adoptive therapy of solid and hematopoietic cancers [1, 5]. However, this approach has encountered several drawbacks including: insufficient cell number, limited donors, lower response to stimulants, decreased expansion potential and increased dysfunctionality. To address these limitations, other sources for NK cells such as bone marrow, embryonic cells, induced pluripotent and umbilical cord blood stem cells (UCB-HSC) have been tested [6–10].

UCB-HSCs have significant advantages over other sources of NK cells including availability, easy expansion and differentiation into other immune cells, containing numerous NK cell progenitors, fast engraftment and immune reconstitution, less stringent requirements for HLA matching and lower risk of graft versus host disease (GvHD); hence, they can be used to generate NK cells [1, 6, 11]. Various methods have been developed to generate NK cells with high purity (> 90%) from UCB-HSCs [6–10]. Although several clinical trials have been performed; it is still difficult to obtain functional NK cells by ex vivo cultivation of UCB-HSCs [6, 7]. The dysfunctional NK cell phenotypes are often determined as down regulation of specific surface activating receptors including NKG2D, CD16 and NCRs (NKp30, NKp44 and NKp46) [12–14] and up regulation of the inhibitory receptors including NKG2A, Tim3 and PD-1 [13, 15–18] on NK cells in tumors and chronic infections [13].

RAS/MAPK, PI3K/AKT, JAK/STAT and TGF-β are among the most prominent signaling pathways involved in switching between NK activation and inhibition [13, 14, 16]. PI3K signaling pathway is activated by the activating receptors signals such as 2B4 and KIR receptors and plays a key role in NK cells activation. Activate MEK-ERK pathway, which is a key signaling pathway for actin reorganization and cell polarization. PI3K signaling pathway is involved in the cytotoxicity and migration of NK cells by controlling actin remodeling, polarization, and even granule exocytosis [12–14]. NK cells also use PI3K-AKT-mTOR pathway to modulate their development, differentiation, and activation. This signaling pathway in NK cells can be stimulated by JAK-STAT pathway and the related cytokines. The signaling pathways have the potential to affect the cytokine priming for NK cell activation by stimulation with common gamma chain cytokines (IL-2, -15, -21) or IL-12 and IL-18 [13, 15]. TGF-β is a negative-regulatory cytokine on NK cells anti-tumor function which can be particularly effective in negatively regulating IFN-γ production and expression of NKG2D and NKp30 activating receptors. TGF-β also alters the surface expression of chemokine receptors such as CXCR3, CXCR4, CX3CR1 and has negative effects on the migration of NK cells to the tumor site [13, 16–18].

Therefore, targeting these pathways may be an effective strategy for producing active and functional NK cells. In the present study to promote NK differentiation, RAS/MAPK pathway was activated using IGF-1 [19], JAK/STAT signaling pathway was induced using IL-21 [20, 21] and TGF-β signaling pathway was blocked using SIS3 [22–24]. Moreover, IL-7 was used as the NK cells differentiation and maturation factor [25, 26].

**Fig. 1:**
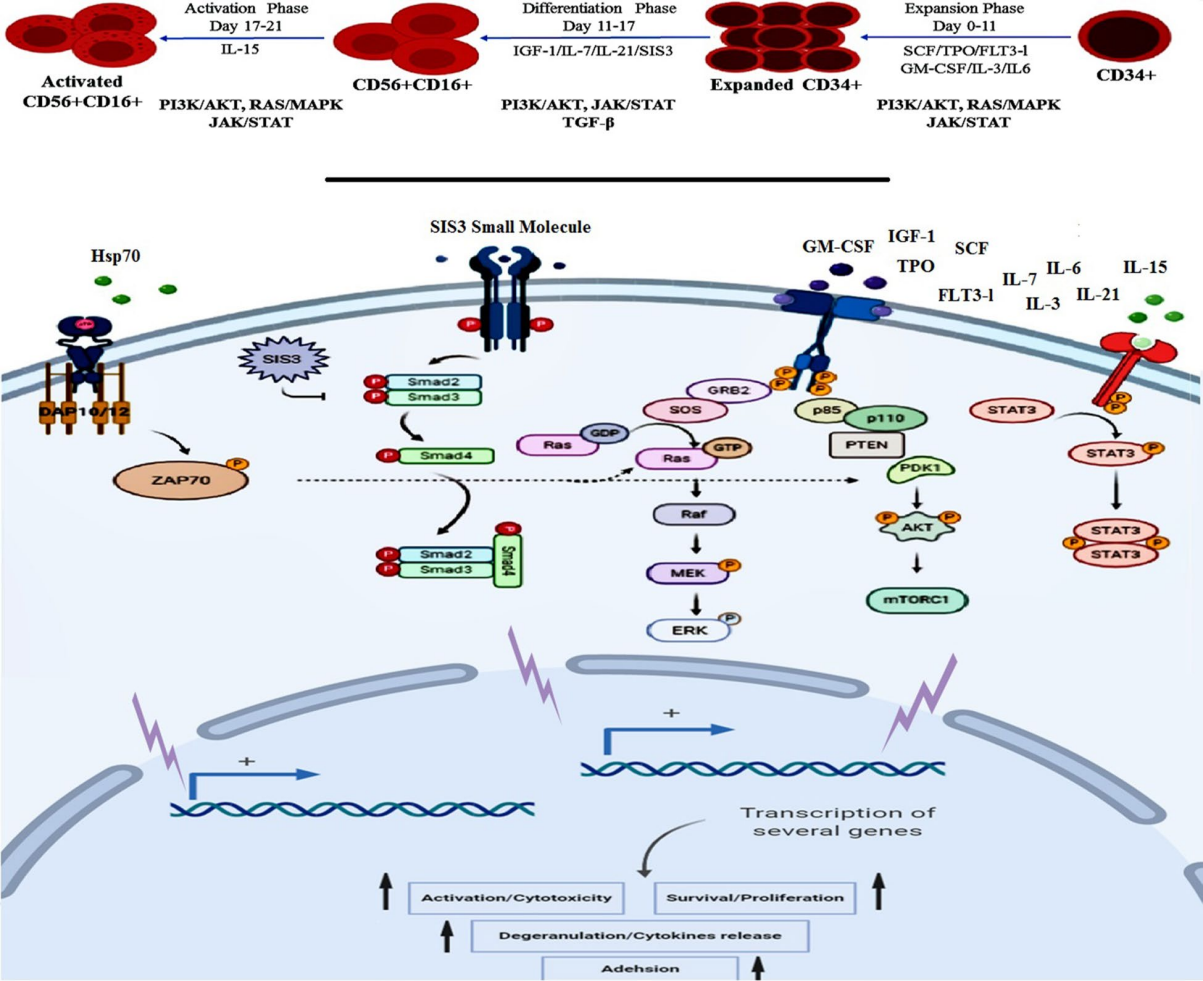
Conceptual schema of the generation and activation of NK cells. In the present study, RAS/MAPK, PI3K/AKT, JAK/STAT pathways (involved in NK cells differentiation and activation) and TGF-β pathway (involved in NK cells dysfunction) were induced and inhibited respectively

The aim of the present study was to differentiated to functional NK cells from UCB-CD34 + through manipulation of RAS/MAPK, PI3K/AKT, JAK/STAT and TGF-β as the critical activation and inhibition signaling pathways of NK cells starting from the early differentiation phase (Fig. 1). Another goal of this study was achieving the desirable NK cell quantity during the differentiation process.

## Methods

### Cell lines

K562 and SKOV3 cell lines (Royan Institute Cell Bank, Tehran, Iran) were cultured in RPMI 1640 (Gibco, USA) containing 50 U/ml penicillin, 50 mg/ml streptomycin and 10% FBS and incubated at 37 °C in 5% CO_2_.

### Isolation and expansion of CD34 + cells

MNCs were harvested from UCB using Ficoll-Hypaque density gradient centrifugation and then CD34 + cells were isolated using magnetic bead selection (Miltenyi Biotech, USA). The purity of the isolated cells was 92% ± 2.30 (Additional file 1: Figure S1) which was confirmed by flowcytometry and specific antibody against CD34.

CD34 + cells were seeded at 7 × 10^5^ cells per well in 6-well plates (TPP, Switzerland) in Advanced RPMI 1640 medium (Gibco, USA) with penicillin/streptomycin (100 U/ml) and cytokines cocktail (SCF, TPO, FLt3-L, IL-3, IL-6, and GM-CSF) (all from R&D Systems, USA) at the expansion phase of cultures (Table 1). Every 2–3 days, half of the media was replaced with fresh media for 7 days. Fold expansion was calculated by dividing the absolute output number of the expanded cells after each phase of culture by the respective number on the day 0.

**Table 1:** Culture media in different phases of NK generation

Phase	Cell culture medium	Concentration (ng/ml)	Exposure time (day)
Expansion	SCF	50	7
	TPO	50	
	FLt3-L	50	
	GM-CSF	50	
	rhIL-3	25	
	rhIL-6	25	
Differentiation	SCF	25	10
	TPO	25	
	rhIGF-1	50	
	rhIL-7	25	
	rhIL-21	25	
	SIS3 (Smad3 inhibitor)	0.125	
Activation	SCF	2.5	4
	TPO	2.5	
	rhIGF-1	5	
	rhIL-7	10	
	rhIL-15	50	
	rhIL-21	10	
	SIS3 (Smad3 inhibitor)	0.125	
	rhHsp70	2000	

SFT (SCF/ FLt3-L/TPO), SFT36G (SCF/ FLt3-L/TPO/IL-3/IL-6/GM-CSF)

I7 (IGF-1/IL-7), I21 (IGF-1/IL-21), I721 (IGF-1/IL-7/IL-21), IS721 (IGF-1/SIS3/ IL-7/IL-21)

### Differentiation of CD34 + cells to NK cells and their activation

To differentiate CD34 + cells into NK cells, the expanded cells at the previous step were seeded at the density of 7 × 10^5^ cells per well in 6-well plates in the NK cell differentiation medium composed of SCF, TPO, rhIGF-1 (Royan Biotech CO, Iran), rhIL7, rhIL21 and SIS3 (Smad3 inhibitor) (Calbiochem, USA). Half of the medium was changed every 2–3 days for about 10 days (from day 7 to 17 of culture).

To activate the differentiated NK cells, an activation medium containing SCF, TPO, rhIGF-1, rhIL7, rhIL21, SIS3 (Smad3 inhibitor), rhIL15 and rhHsp70 (according to our previous study [27]) was used. The details of culture media used in each phase are listed in table 1.

The fold expand of cells was calculated and their immunophenotype was assessed using specific antibody for each step and by flow cytometry.

### Colony forming unit assay (CFU assay)

The CFU assay was performed using the commercially available methylcellulose medium (MethoCult H4435 Enriched; Stem cell Technologies, Vancouver, BC, Canada). This medium supports the clonal progeny of a single-cell to differentiate and grow in distinct colonies including BFU-E (burst-forming unit-erythroid), CFU-E (colony-forming unit-erythroid), CFU-GEMM (colony-forming unit-granulocyte, erythroid, macrophage, megakaryocyte) and CFU-GM (colony-forming unit-granulocyte, macrophage). 1 × 10^3^ CD34+ cells were added to 2 ml MethoCult H4435 medium and vortexed vigorously until the cells were well suspended and 1 ml of the cell suspension was subsequently plated in a 12-well plate at a final density of 5 × 10^2^ cells/well. Cells were finally incubated at 37 °C in 5% CO_2_ for 12–14 days.

### Wright–Giemsa staining

Morphological characteristics of the expanded CD34 + cells were detected by Wright–Giemsa staining on the day 14. Briefly smears of cells were stained with Wright–Giemsa solution for 25 min, rinsed with distilled water and air dried and cells morphology was finally studied by light microscopy.

### Flowcytometry analysis

Single cells at each step were labeled with specific antibodies including anti-hCD45-FITC, anti-hCD34-PE, anti-hCD3-FITC, anti-hCD56/CD16-PE, anti-hNKG2D-PE, anti-hNKG2A-Alexa Fluor488, anti-hNKp30-PE and anti-hNKp44-Alexa Fluor488 (all from BD Biosciences, USA) as multicolor staining and then analyzed using BD FACS Calibure. The data was further analyzed using FlowJo software Ver.10.6.1. The percentage of CD34 + cells was assessed in expansion phase while the percentage of CD3 − CD56 + /CD16 + cells was tested in the differentiation phase. In the activation phase, the expression percentage of CD3 − CD56 + /CD16 + , NKG2D + , NKG2A + , NKp30 + and NKp44 + markers on NK cells were assessed.

### Quantitative real time reverse transcription PCR (Q-RTPCR)

RNAs were isolated using Gene All Hybrid-R kit (Biotech, Korea). Spectrophotometric methods were used to assess the quality and quantity of RNAs. cDNA was synthesized from 1 μg of total RNA with RT-for-PCR kit (Takara Bio, Inc., Otsu, Japan) according to the manufacturer’s instructions. The sequence of primers used for measuring gene expression is listed in Additional file 4: Table S1. β2-microglubolin RNA levels was used as an internal control for all experiments. Amplification was performed for 1 cycle of a sequential incubation at 50 °C for 2 min, 95 °C for 10 min, and subsequent 40 cycles of a consecutive incubations at 94 °C for 1 min, at 60 °C for 1 min and at 72 °C for 1.5 min. The individual gene expression value was calculated after normalization to β2-microglubolin gene. Quantitation of the mRNAs was determined by the comparative cycle threshold (CT) method. The relative change in mRNA concentration for each test sample was then determined from the difference between the calibrator CT and the CT of each test sample. Each sample was run in triplicate and the relative expression of the target genes was determined using 2^−ΔΔCt^ equation.

Perforin, IFN-γ, Granzyme A and B mRNA levels were evaluated by quantitative real-time RT-PCR (SYBR Green assay) using a Rotor-Gene 6000 (Corbett Life Sciences, Sydney, Australia) Sequence Detector.

### Cytotoxicity assay

K562 (chronic myeloid leukemia) and SKOV3 (malignant ovarian cancer line) cells were inactivated with Mitomycin C (10 μg/ml) to inhibit their proliferation, then they were labeled with Calcein AM for 45 min incubation at 37 °C in 5% CO_2_. The labeled cells were washed with PBS, re-suspended in RPMI with 10% FBS, and plated in 6-well plates at 3 × 10^5^ cells/well in triplicate as target cells. NK cells as the effector cells were co-cultured with K562 cells at effector:target (E:T) ratios of 3:1 and 5:1 followed by incubation for 24, 48 and 72 h at 37 °C in 5% CO_2_. For SKOV3 cells we used 3:1 E:T ratio and incubated for 48 h at 37 °C in 5% CO_2_. Cytotoxicity level was evaluated by calcein/propidium iodide (PI) staining and flowcytometry. The control groups were the tumor cells cultivated in the same medium, which were not treated with NK cells.

### Statistical analysis

The statistical analysis was performed using Graph pad Prism Ver.8 and the data was presented as the means ± SD. Statistical differences were determined using paired Student’s t tests or two‐tailed Student’s t tests when comparing two groups and one-way or two‐way ANOVA analysis when comparing more than two groups. Differences were considered statistically significant at P < 0.05. Data from three different biological replicates was collected.

## Results

### SFTG36 as the proper cytokine combination for the expansion of UCB hematopoietic CD34 + cells

**Fig. 2:**
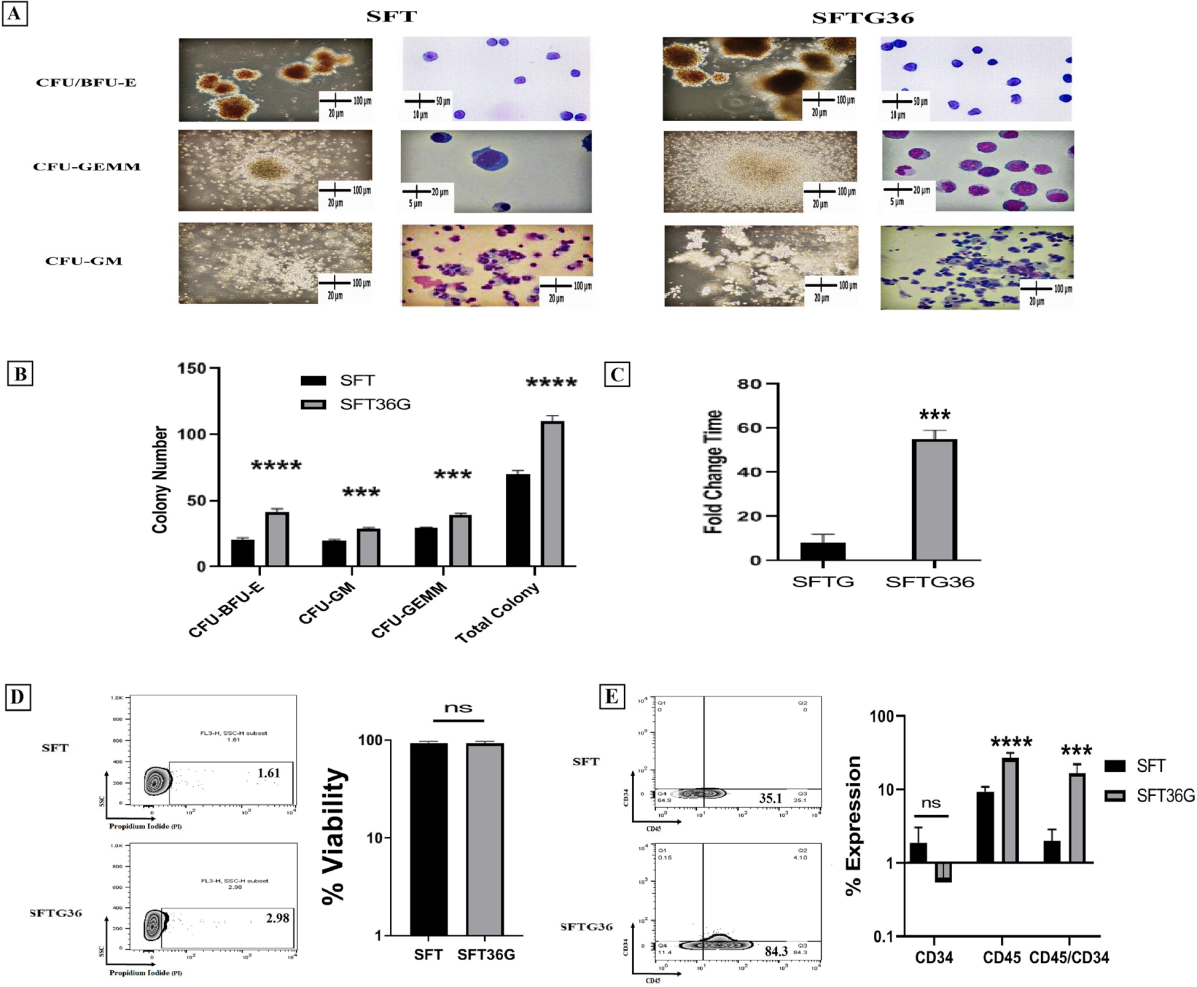
Characterization of the expanded HSCs CD34+ progenitor. **A** and **B**, the comparison of CFU assay results between the two groups treated with SFT (SCF, Flt3-L and TPO) and SFTG36 (SCF, Flt3-L, TPO, GM-CSF, IL-3 and IL-6) cocktail cytokines and morphological analysis by Wright-Giemsa staining. **C**–**E**, both expanded cell populations were analyzed using flowcytometry and cell counting on the day 7. **C**, Analyses of fold expansion and D, cell viability of both groups. **E**, the phenotypic analysis of the expanded cells for CD45, CD34 and CD45/CD34 markers are shown as flowcytometry zebra plots. The comparison of markers expressions ****P < 0.0001, indicating a statistically significant increase in the expanded SFTG36 cell population compared to SFT cell population. p<0.01 was considered significant (*P ≤ 0.05, **P ≤ 0.01, ***P ≤ 0.001 and ****P ≤ 0.0001). Data are demonstrated as mean ± SD, n =4.

As mentioned in the method section, we differentiated and activated NK cells in a three-step protocol; expansion, differentiation and activation phases. As the first step, we sought to determine the best cocktail of cytokines for CD34 + cells expansion. Different combination of cytokines was selected based on literature and divided into two groups: SFTG36 medium containing SCF/FLt3-L/TPO/GM-CSF/IL-3/IL-6 and SFT medium containing SCF/FLt3-L/TPO. Our results indicated that a higher number with larger colonies of CFU-BFU-E, CFU-GM and CFU-GEMM was formed in CD34 + cells treated with SFTG36 (P < 0.0001) (Fig. 2A, B). The obtained results were also confirmed morphologically through Wright–Giemsa staining (more lymphoid progenitors) (Fig. 2A). The fold expansion was 53.72 ± 1.86 fold in SFTG36 and 8.00 ± 3.98 fold in SFT media after 7 days of cultivation (Fig. 2C). The cell viability in both groups was higher than 95% (Fig. 2D). Moreover, 79.55 ± 18.2% and 42.33 ± 19.05% of cells in SFTG36 was CD45 + and CD45 + /CD34 + cells, that was higher compared to SFT group (19.52 ± 4.276% CD45 + and 2.5 ± 1.51% CD45 + /CD34 +) (Fig. 2E). Therefore, based on the above data, the cells expanded in SFTG36 medium were considered for NK differentiation.

### The significant roles of Smad3 blockade and IGF-1 in NK cell differentiation

As mentioned earlier, different signaling pathways affect NK cell differentiation and the efficacy of the differentiation procedure. In the present study, we used IGF-1 to activate RAS/MAPK pathway, IL-21 to induce JAK/STAT signaling pathway, SIS3 to block TGF-β signaling pathway, and IL-7 as NK cells differentiation and maturation factor. Therefore, the expanded HSCs were cultivated in I721 medium containing IL-7/IL-21 + IGF-1 and IS721 medium containing IL-7/IL-21 + IGF-1 + SIS3.

**Fig. 3:**
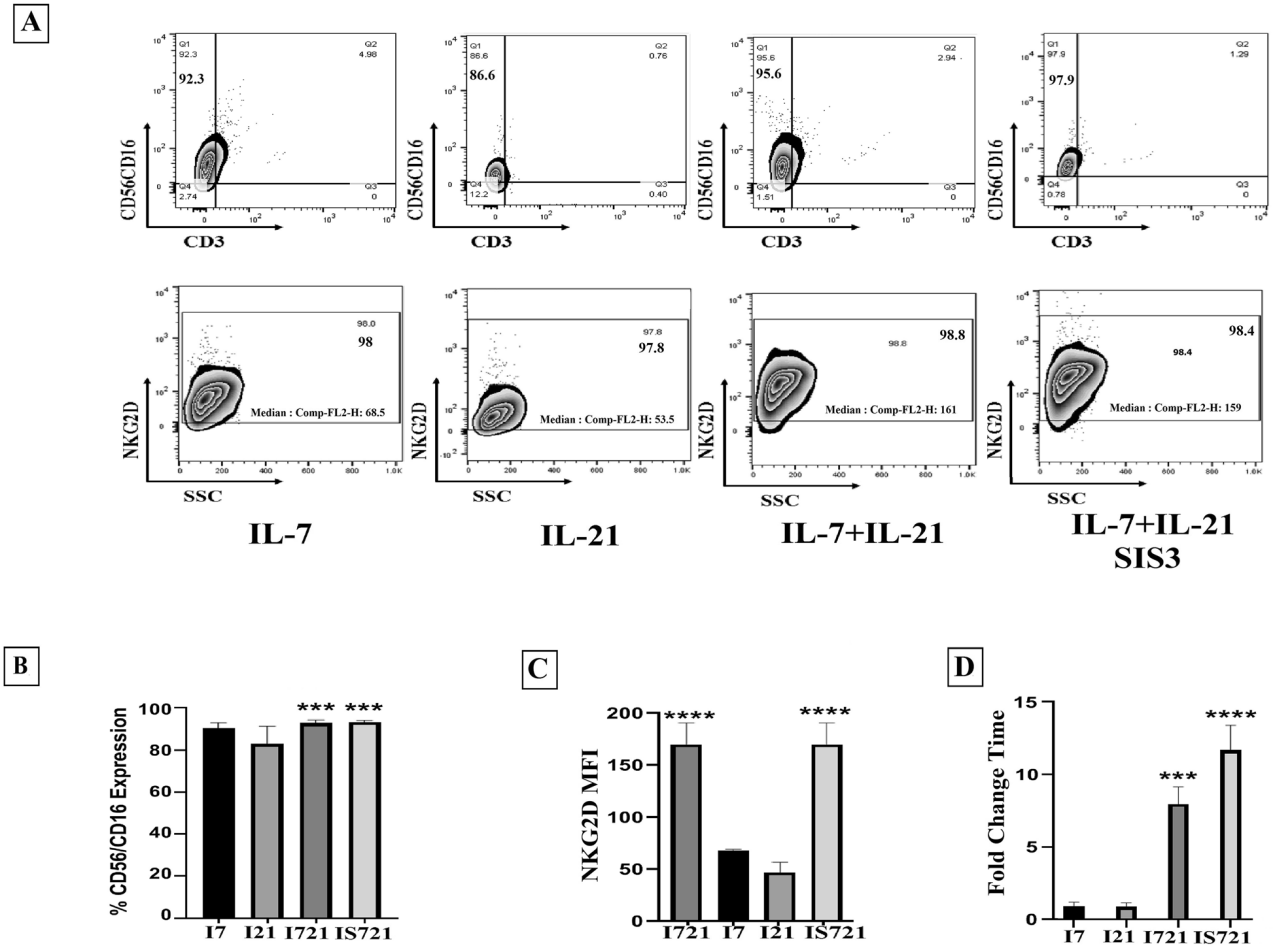
Comparison of characteristics of differentiated NK cells. **A** and **B** assessment of CD56/CD16 and CD3 markers expression among all 4 groups (IL-7+IGF-1, IL-21+IGF-1, IL7+IL-21+IGF-1 and IL-7+IL-21+SIS3+IGF-1) showed a significant expression difference in the groups IL-7+IL-21+ IGF-1 (*P* < 0.01) and IL-7+IL-21+ IGF-1+SIS3 (*P* < 0.01). **C**, these 2 groups also demonstrated significant difference in the expression of NKG2D compared to the other groups. **D**, the expansion potential analysis showed that IL-7+IL-21+ IGF-1+SIS3 group had the highest level of Fold expansion among all groups (*P* < 0.05). It should be noted that all expressions were assessed in the viable cells (>90%±3.00). *P*<0.01 was considered significant (*P ≤ 0.05, **P ≤ 0.01, ***P ≤ 0.001 and ****P ≤ 0.0001). Data is demonstrated as mean ± SD, n = 3.

Our results indicated a low percentage of T cells (CD3 +) and NKT cells (CD3 + /CD56 + CD16 +) in all tested groups (< 1% and < 2%, respectively) following 17 days of cultivation (Additional file 2: Figure S2). Both differentiation media, represented the highest percentage of CD56 + 16 + CD3 −  cells (92.7% ± 1.45 in I721 and 93.23% ± 0.75 in IS721 group) (Fig. 3A, B). the expression of NKG2D activating marker also demonstrated a significant increase in MFI (168.00 ± 20.88 in I721 and 168.66 ± 20.08 in IS721 group) (Fig. 3A, C; Additional file 2: Figure S2). The fold expansion of the differentiated cells was assed and the cells treated with I721 (8.093 ± 1.205) and IS721 demonstrated a more significant expansion (11.893 ± 1.712) compared to the other group (Fig. 3D). Finally, the cells differentiated in IS721 medium based on phenotyping and proliferative potential were subjected for NK activation.

### Evaluation of the induced NK cell function

**Fig. 4:**
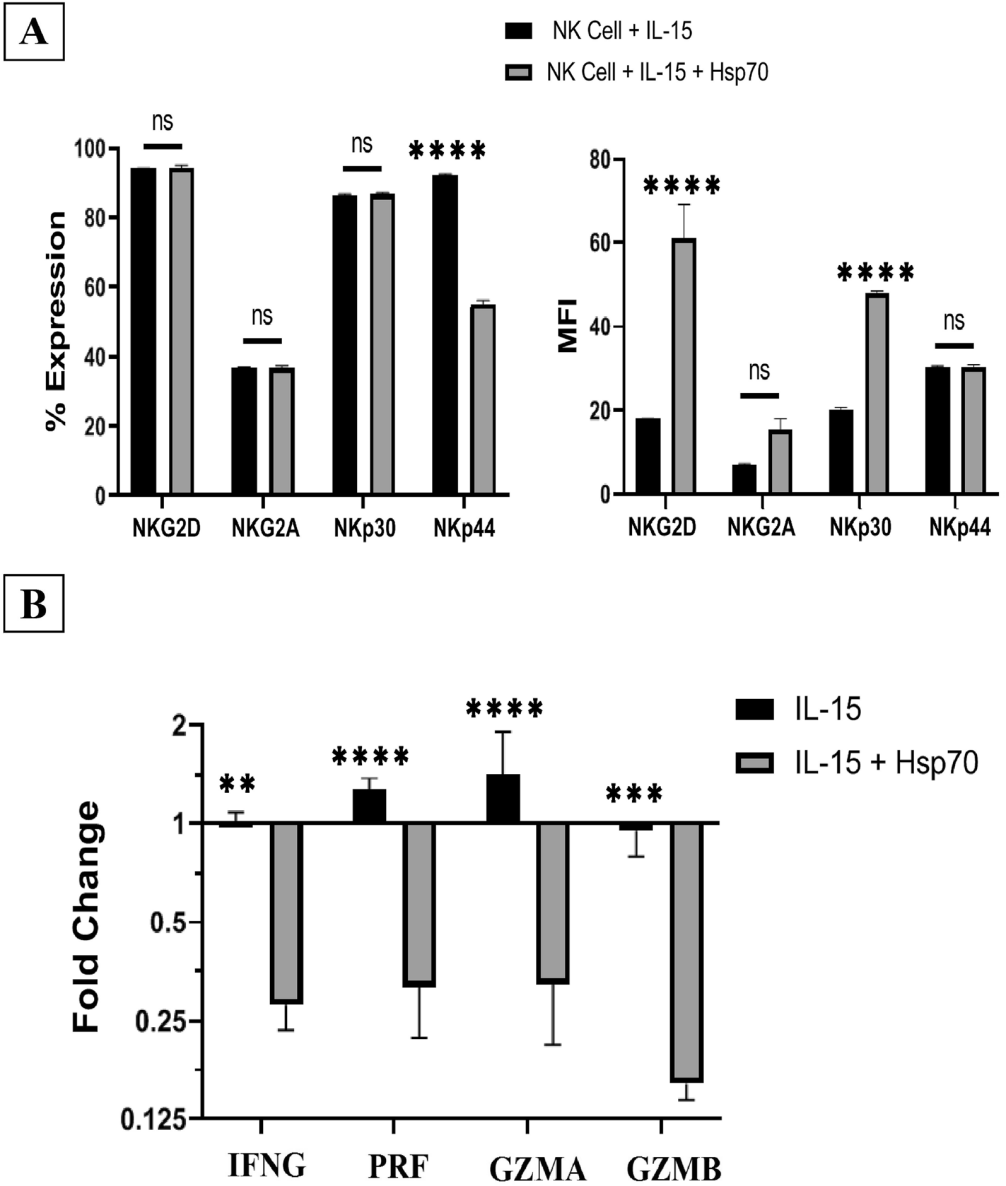
Comparison of characteristics of the activated NK cells. **A** Immunophenotyping of activated NK cells (n = 5). **B** Analysis of the quantitative gene expression differences of Perforin, IFN-γ, Granzyme A and B between IL-15 and IL-15/Hsp70-activated NK cells on the 21st day (n = 3). *P* < 0.01 was considered significant (*P ≤ 0.05, ** *P* ≤ 0.01, *** *P* ≤ 0.001 and **** *P* ≤ 0.0001). Data is demonstrated as mean ± SD

To evaluate the function of the differentiated NK cells we entered them into the activation phase and they were incubated with IL-15 and IL-15/Hsp70, for an additional four days. The potential of the activated NK cells was tested through Immunophenotyping analysis. Both activation groups could increase the percentage of NKG2D, NKG2A and NKp30 positive cells (P > 0.05) (Fig. 4A-left). However, MFI of NKG2D (61.12 ± 17.62, *P* < 0.0001) and NKp30 (48.17 ± 0.83, *P* < 0.0001) were higher in IL-15/Hsp70 -treated group compared to IL-15 treated group (Fig. 4A-right). The expression of perforin (0.317 ± 0.095 fold, *P* < 0.0001), IFN-γ (0.280 ± 0.046 fold, *P* < 0.01), granzyme A (0.323 ± 0.112 fold, *P* < 0.0001) and granzyme B (0.160 ± 0.017 fold, *P* < 0.001) was up regulated in IL-15/ Hsp70 group compared to IL-15 group (Fig. 4B). Meanwhile IL-15 increased the percentage of NKp44 (92.2% ± 0.30) (Fig. 4A-left).

**Fig. 5:**
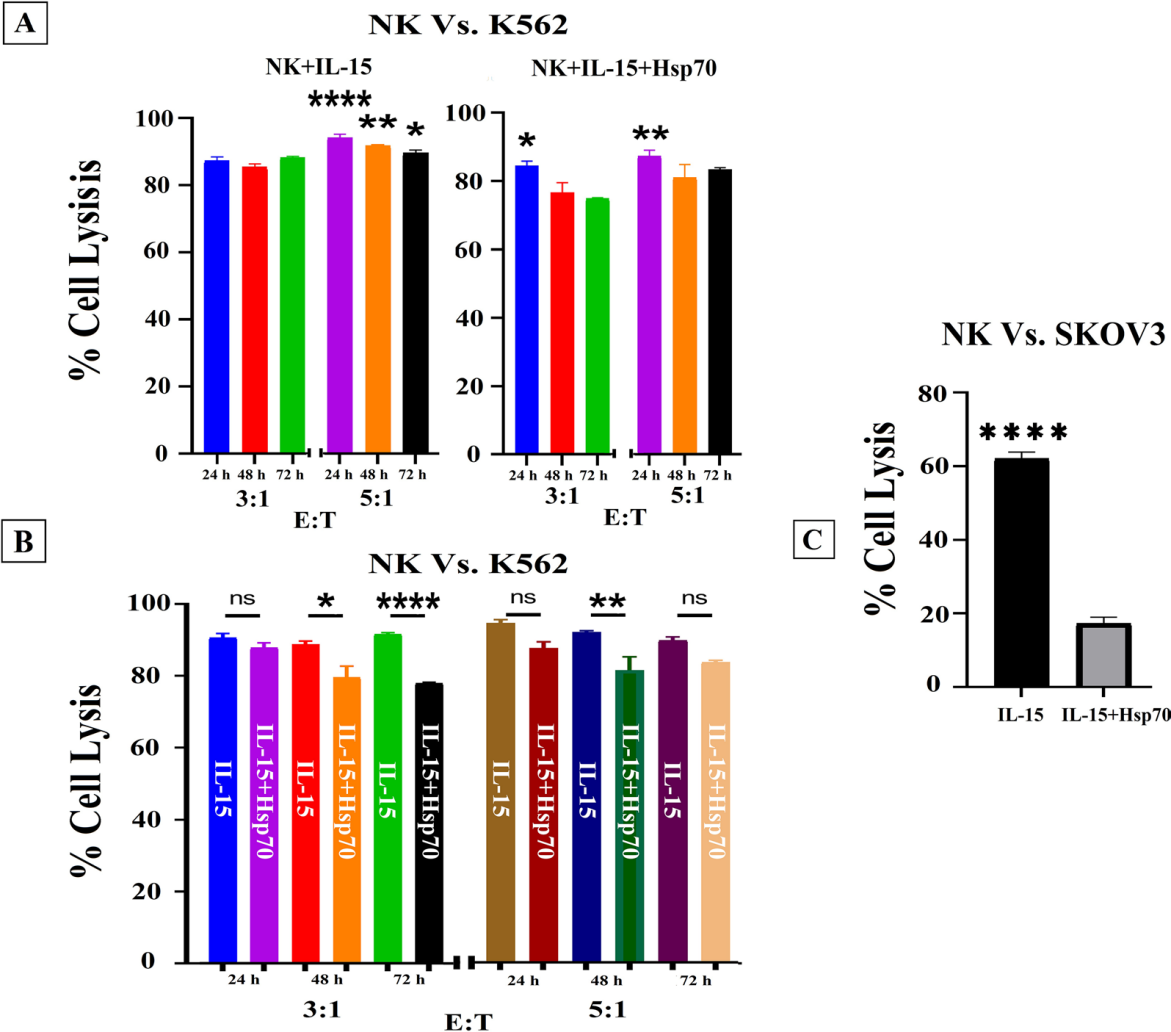
Cell lysis activities of NK cells. **A**, Comparison of the groups at different times and different ratios against K562 cells (n = 4). **B**, Specific cell lysis analysis of IL-15 and IL-15/Hsp70-activated NK cells against K562 cells at different times and effector ratios (n = 4). **C**, Cytotoxicity assay of IL-15 and IL-15/Hsp70-activated NK cells at 3:1 ratio 48 hours after co-culture against SKOV3 cells (n = 7). The control cancer cells viability was about 90%. Data is demonstrated as mean ± SD, *P* > 0.05 which indicates no statistical significance.

The cytotoxic function of NK cells against K562 erythroleukemia cells was surveyed as the gold standard of NK cells activity assay. To perform this assay, NK cells activated by the above mentioned activating combinations, were co-cultured with K562 in 3:1 and 5:1 E:T ratios and the cell lysis potential of NK cells was analyzed in 24, 48 and 72 h following the co-culture (Fig. 5A, B). according to the results of NK cell cytotoxicity assay in the two groups, the highest cell lysis ability was reported in 5:1 ratio and 24 h after co-culture with K562 tumor cells, as 94.22 ± 1.93 (Fig. 5A-left, B) and 87.3 ± 3.45 (Fig. 5A-right, B) in the groups treated with IL-15 and IL-15/Hsp70 respectively. Comparing the two treated groups with each other, the highest cell cytotoxicity was shown to be in the same ratio and time for IL-15 treated group (Fig. 5A, B).

The cytotoxic capacity of the NK cells treated with IL-15 and IL-15/Hsp70 against SKOV3 tumor cells was also assessed as a solid tumor model. According to our data, 3:1 ratio and 48 h led to the highest level of cell lysis in the two groups; however, the cells treated with IL-15 showed significantly higher levels of lysis (> 62%, *P* < 0.0001) compared to the group treated with IL-15/Hsp70 (> 17.27%, *P* < 0.001) (Fig. 5C; Additional file 3: Figure S3). Finally, it was observed that the differentiated NK cells can retain their cytolytic potential and play their antitumor role for up to 72 h post co-culture.

## Discussion

In the present study, a highly efficient cell culture protocol for ex vivo generation of functional NK cell products from UCB-HSCs through targeting RAS/MAPK, PI3K/AKT, JAK/STAT and TGF-β pathways was demonstrated which led to their enhanced functional activity and proliferation (Fig. 1). The cells obtained in this study have significant expansion potential of more than 600 folds (53 folds in the expansion phase and 12 folds in the differentiation phase) (Figs. 2C, 3D) due to the use of lower doses of the molecules including 2.5 ng/ml SCF, 2.5 ng/ml TPO, 5 ng/ml rhIGF-1, 10 ng/ml rhIL7, 10 ng/ml rhIL21, 25 ng/ml SIS3 inhibitor, 50 ng/ml rhIL15 and 20 µg/ml rhHsp70 compared to other studies (SCF 100 ng/ml and TPO 100 ng/ml) [28–32]. Increasing and maintaining the proliferation capacity and the cell lysis potential of NK cells were the main objectives of the present study and to achieve these goals, RAS/MAPK, PI3K/AKT, JAK/STAT and TGF-B pathways were manipulated with low doses of SFTG36/IS721/IL-15 (Fig. 1). In this study, the differentiated NK cells displayed high expression of CD56 (> 95.00%) and several activating receptors such as NKG2D (> 94.00%), NKp30 (> 86.00%) and NKp44 (> 86.00%); while the expression of NKG2A (< 36.00%) inhibitory receptor was not elevated (Figs. 3B, C; 4A).

To date, various protocols have been employed for ex vivo expansion and differentiation of NK cells from HSC sources. Despite using different combinations of cytokines with or without feeder cell lines, and the use of animal and human sera, several challenges remain still to be overcome, including the inefficiency of NK cell products [7, 9, 33–37].

It has been shown that the expanded CD34 + cells could be differentiated into NK cell with an average purity of 40–60% after 5–7 weeks of culture [7, 9, 34, 35]. Many studies have reached a calculated mean expansion rate of 300-fold during the NK cell generation phase.

Several studies have reported a multiple cytokines-based culture method for up to 6 weeks to generate the therapeutic NK cell products with high cytotoxicity function (10:1, 85%) from CB-derived CD34 + HSCs [8, 9, 35]. In the present study, a 600-fold expansion of NK cells with purity of  > 90% was demonstrated after 3 weeks of culture and the NK cells differentiated from UCB-CD34 + HSCs were assayed in 3:1 and 5:1 ratios so as to measure the cytotoxicity potential. These cells differentiated with a growth factor cocktail demonstrated cell lysis of  > 90% against K562 tumor cells and  > 60% against SKOV3 tumor cells (Fig. 5).

Generation of functionally mature killer cells is one of the fundamental problems encountered during the differentiation and activation of NK cells. In most studies, the media containing IL-2 are used for NK cells differentiation and activation [12, 13, 20, 38]. Functionality can be induced using exogenous molecules regulating the NK cells effector factors such as IL-2, IL-15 and IL-21. They do so by increasing the expression of co-inhibitory receptors (PD-1), inhibitory receptors (NKG2A) and the upregulation of the activating receptors like NKG2D.All these changes can impair the balance of activating/inhibitory signals and consequently result in the exhaustion of NK cell functions [20, 21, 27, 39–41]. Other factors that can suppress NK cells function during differentiation and activation are suppressive cytokines such as TGF-β which induces the downregulation of activating receptors [12, 13, 20, 40, 42]. The results of this study indicated that the differentiated NK cells have low level of NKG2A inhibitory receptors which can be the reason why the cells function equally against K562 cells [12, 13, 42].

In addition, Hsp70 in the previous studies have been shown to increase the induction of NK cells. The declined cytotoxic function of Hsp70-treated NK cells against K562 and SKOV3 cells in the present study (Fig. 5; Additional file 3: Figure S3) can be explained by the dual function of Hsp70 which can also promote the cancer cells viability [27, 43–47]. Furthermore, considering the qRT-PCR results, it can be claimed that treating with Hsp70 led to a reduction in the NK cell lysis capacity compared to the other group.

A previous study has reported the positive effect of IGF-1 on human NK cell cytotoxicity and assessed the ability of endogenous miR-483-3p to regulate IGF-1 expression in UCB HSCs -derived NK cells using anti-miR-483-3p to upregulate IGF-1 mRNA and protein levels. However, the exact regulatory mechanism of IGF-1 in enhancing NK cell function remains unclear. It was previously shown that IGF-1 promotes the cytotoxic function of UCB HSCs-derived NK cells [19]. But in this study, rhIGF-1 protein was used as an exogenous treat in 50 ng/ml for 14 days to induce insulin receptor-dependent pathways (RAS/MAPK and PI3K/AKT), and the results showed that this growth factor was effective in NK cells proliferation, maturation and activation.

In conclusion, the obtained results in the present study show that the NK cells derived via our method can induce significant lytic function against K562 (> 90%) and SKOV3 (> 60%) targets in the lower ratio of effector cells compared with other studies (5:1), suggesting that the ex vivo generated UCB HSCs -derived NK cells could be suitable candidate for designing adoptive NK cell-based therapy for cancer (Fig. 5; Additional file 3: Figure S3).

This study is the first to use the combination of rhIGF-1 protein and TGF-B blockade to generate cytotoxic NK cells, and further studies are required on the other tumor cells as well as on the in vivo models. It is also essential to optimize the activation pathways and compare the cell lysis activities between CB-derived NK cells and the peripheral blood-derived NK cells.

## Supplementary Information

**Additional file 1:** Figure S1. CD34+ cells sorting by MACS.

**Additional file 2: **Figure S2.Differentiated-NK cells phenotype characterization

**Additional file 3:** Figure S3.Cytotoxicity of differentiated- NK cells against SKOV3 cells

**Additional file 4: Table S1.** Culture media in different phases of NK generation.

## Data Availability

The datasets used and analyzed during the current study are available from the corresponding author on reasonable request.

## Abbreviations

CML: Chronic myeloid leukemia
UCB: Umbilical cord blood
E:T: Effector-to-target
HSCs: Hematopoietic stem cells
IL: Interleukin
MNCs: Mononuclear cells
NK: Natural killer
