# Supplementary figures and images for: Overcoming the UCB HSCs –Derived NK cells Dysfunction through Harnessing RAS/MAPK, IGF-1R and TGF-β Signaling Pathways
Source: Cancer Cell Int. 2021 Jun 7;21:298. doi: 10.1186/s12935-021-01983-z (PMC8185927; doi:10.1186/s12935-021-01983-z)

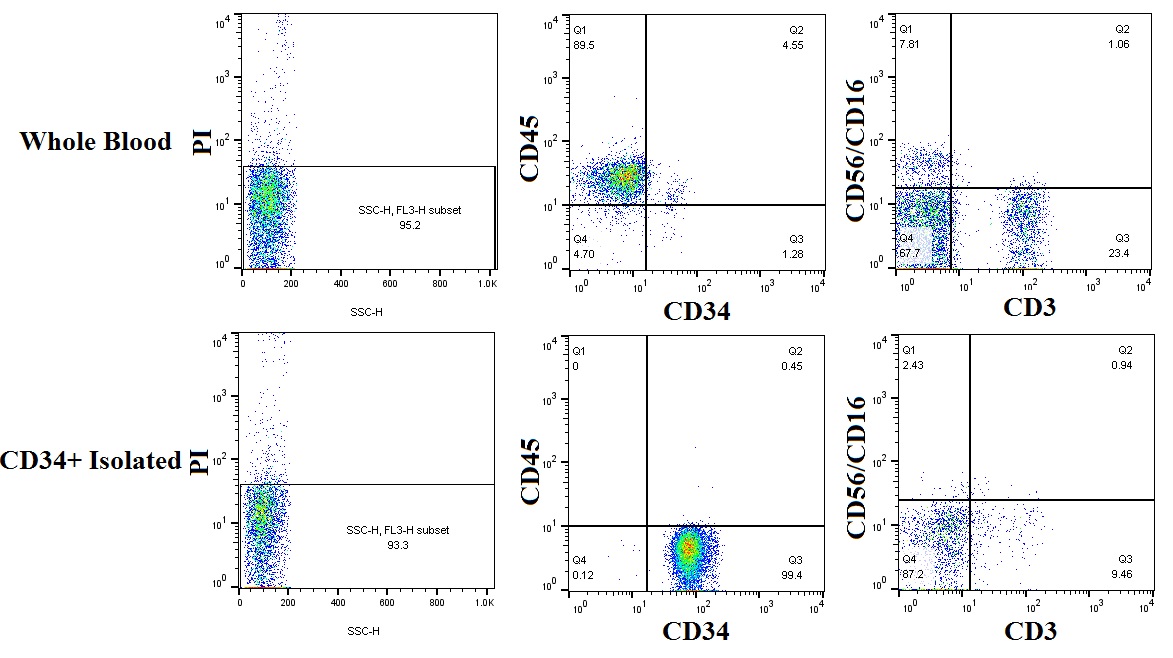

Supplement: Supplementary file 1 — Additional file 1: Figure S1. CD34+ cells sorting by MACS. [file 12935_2021_1983_MOESM1_ESM.jpeg]

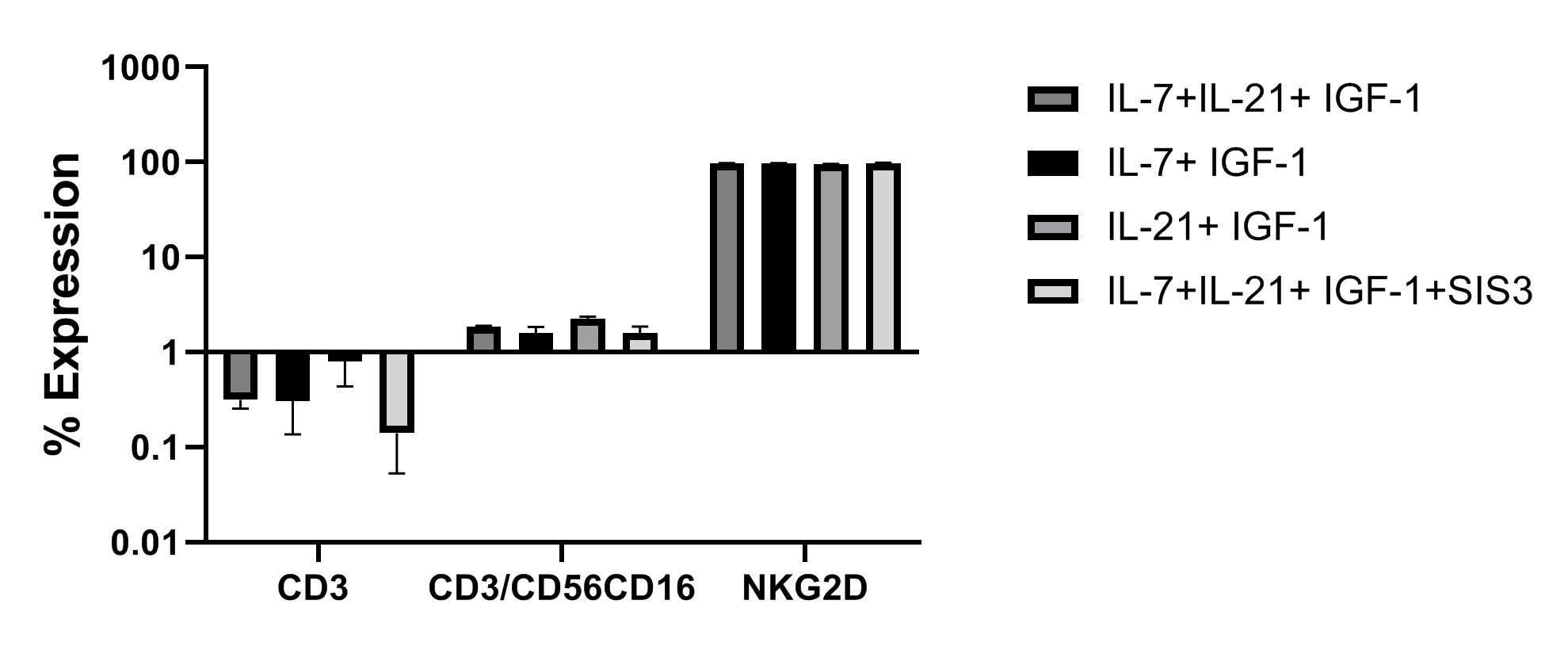

Supplement: Supplementary file 2 — Additional file 2: Figure S2.Differentiated-NK cells phenotype characterization [file 12935_2021_1983_MOESM2_ESM.jpeg]

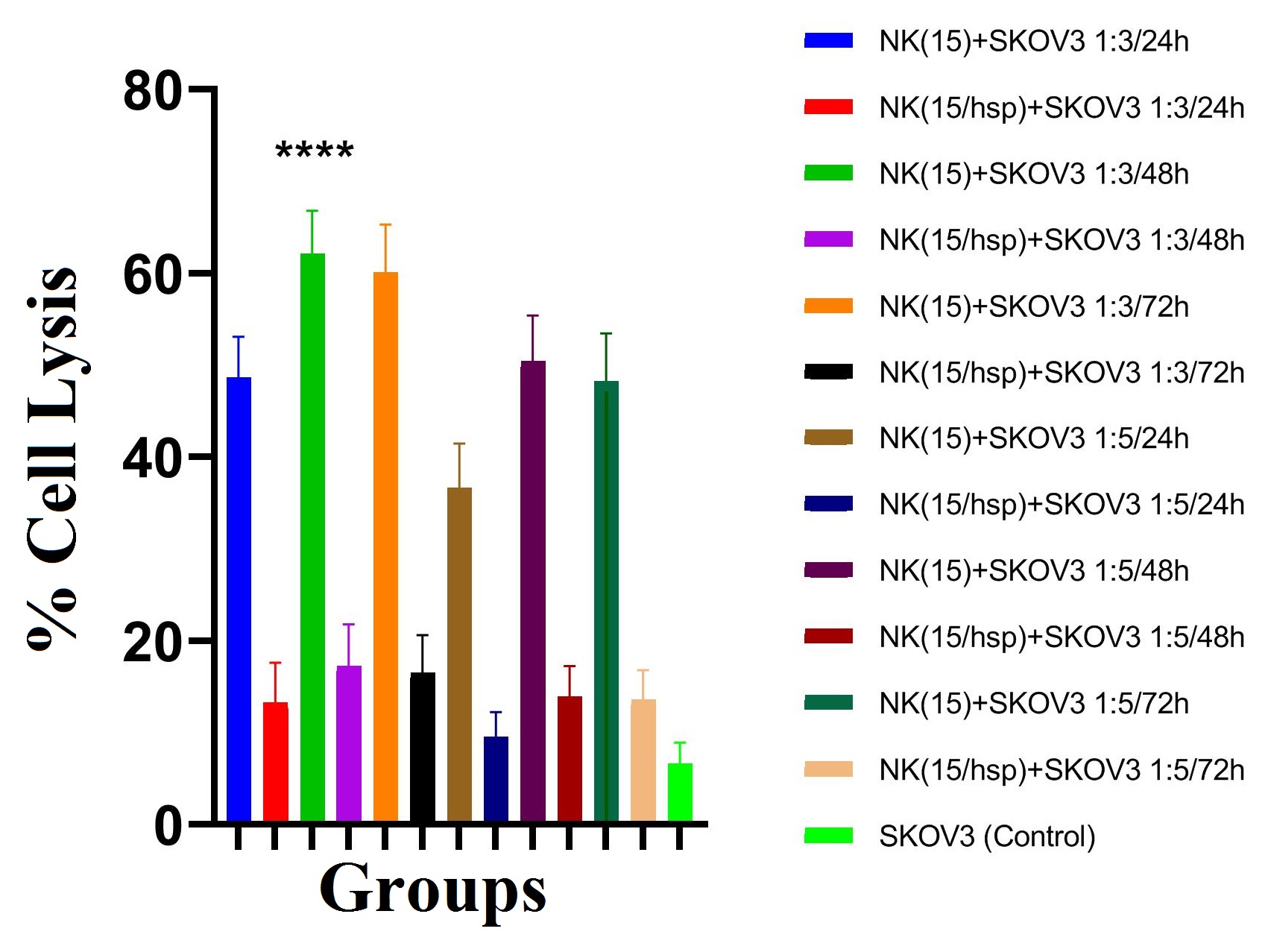

Supplement: Supplementary file 3 — Additional file 3: Figure S3.Cytotoxicity of differentiated- NK cells against SKOV3 cells [file 12935_2021_1983_MOESM3_ESM.jpeg]
