# Supplementary material for: Overcoming the UCB HSCs –Derived NK cells Dysfunction through Harnessing RAS/MAPK, IGF-1R and TGF-β Signaling Pathways
Source: Cancer Cell Int. 2021 Jun 7;21:298. doi: 10.1186/s12935-021-01983-z (PMC8185927; doi:10.1186/s12935-021-01983-z)
Supplement: Supplementary file 4 — Additional file 4: Table S1. Culture media in different phases of NK generation. [file 12935_2021_1983_MOESM4_ESM.docx]

**Supplementary Table 1.** Real-time PCR primers description

| **Gene** | **GenBank accession number** | **Primer sequences** | **Amplicon size** | **Annealing temperature** |
| --- | --- | --- | --- | --- |
| **IFN-g** | [NM_000619.3](https://www.ncbi.nlm.nih.gov/entrez/viewer.fcgi?db=nucleotide&id=1519243198) | F: 5'- AAGTGATGGCTGAACTGTCG-3' | 105 bp | 59°C |
|  |  | R: 5'- GCAGGCAGGACAACCATTAC-3' |  |  |
| **Perforin** | [NM_005041.6](https://www.ncbi.nlm.nih.gov/entrez/viewer.fcgi?db=nucleotide&id=1890333443) | F: 5'-CGCCTACCTCAGGCTTATCTC-3' | 155 bp | 58°C |
|  |  | R: 5'-CCTCGACAGTCAGGCAGTC-3' |  |  |
| **Granzyme B** | [NM_004131](https://www.ncbi.nlm.nih.gov/nuccore/NM_004131) | F: 5'-TGGGGGACCCAGAGATTAAAA-3' | 100 bp | 55°C |
|  |  | R: 5'-TTTCGTCCATAGGAGACAATGC-3' |  |  |
| **Granzyme A** | [NM_006144.4](https://www.ncbi.nlm.nih.gov/entrez/viewer.fcgi?db=nucleotide&id=1519315261) | F: 5'- TCAGGTTGATTGATGTGGGACAG -3' | 163 bp | 62°C |
|  |  | R: 5'- GACCATGTAGGGTCTTGAATGAGGA -3' |  |  |
| **B2M** | [NM_004048.4](https://www.ncbi.nlm.nih.gov/entrez/viewer.fcgi?db=nucleotide&id=1825761146) | F: 5'-AGATGAGTATGCCTGCCGTG -3' | 96 bp | 60°C |
|  |  | R: 5'- TCAAACCTCCATGATGCTGCT -3' |  |  |

- IFNγ (IFNG), Perforin (PRF), Granzyme (GZM), β2-microglubolin (B2M)
